# Recent Multiomics Approaches in Endometrial Cancer

**DOI:** 10.3390/ijms23031237

**Published:** 2022-01-22

**Authors:** Dariusz Boroń, Nikola Zmarzły, Magdalena Wierzbik-Strońska, Joanna Rosińczuk, Paweł Mieszczański, Beniamin Oskar Grabarek

**Affiliations:** 1 Department of Histology, Cytophysiology and Embryology, Faculty of Medicine, University of Technology in Katowice, 41-800 Zabrze, Poland; nikola.zmarzly@gmail.com (N.Z.); rektorat@wst.pl (M.W.-S.); 2 Department of Gynecology and Obstetrics with Gynecologic Oncology, Ludwik Rydygier Memorial Specialized Hospital, 31-826 Kraków, Poland; 3 Department of Gynecology and Obstetrics, Faculty of Medicine, University of Technology in Katowice, 41-800 Zabrze, Poland; 4 Katedra Ošetrovatel’stva, Fakulta Zdravotníckych Odborov, Prešovská Univerzita v Prešove, Partizánska 1, 08001 Prešov, Slovakia; joanna.rosinczuk@umed.wroc.pl; 5 Department of Nervous System Diseases, Department of Clinical Nursing, Wroclaw Medical University, 50-367 Wroclaw, Poland; 6 Hospital of Ministry of Interior and Administration, 40-052 Katowice, Poland; pmieszczanski@op.pl

**Keywords:** endometrial cancer, multiomics, multi-omics, biomarkers, diagnostics

## Abstract

Endometrial cancer is the most common gynecological cancers in developed countries. Many of the mechanisms involved in its initiation and progression remain unclear. Analysis providing comprehensive data on the genome, transcriptome, proteome, and epigenome could help in selecting molecular markers and targets in endometrial cancer. Multiomics approaches can reveal disturbances in multiple biological systems, giving a broader picture of the problem. However, they provide a large amount of data that require processing and further integration prior to analysis. There are several repositories of multiomics datasets, including endometrial cancer data, as well as portals allowing multiomics data analysis and visualization, including Oncomine, UALCAN, LinkedOmics, and miRDB. Multiomics approaches have also been applied in endometrial cancer research in order to identify novel molecular markers and therapeutic targets. This review describes in detail the latest findings on multiomics approaches in endometrial cancer.

## 1. Introduction

Endometrial cancer (EC) is the most common gynecological cancer in developed countries. According to the GLOBOCAN database, in 2020, there were 417,367 new cases and 97,370 deaths due to EC worldwide [1]. Endometrial cancer affects mainly postmenopausal women, most often in the sixth and seventh decades of life; however, it is estimated that up to 25% of cases are diagnosed before the menopause [2,3]. Typically, two types of endometrial cancer are distinguished, with type I accounting for the majority of all EC cases. Type I includes the endometrioid EC, which has a good prognosis and is estrogen-dependent. In contrast, type II includes non-endometrioid EC and high-grade EC with poor prognosis [2].

In recent years, due to the use of array- and sequencing-based technologies, an additional EC classification into four genomic groups has emerged: polymerase ε (POLE) ultra-mutated, microsatellite instability (MSI), copy number low, and copy number high [4]. According to the recommendations for the management of patients with EC by the European Society of Gynecological Oncology (ESGO), the European Society for Radiotherapy and Oncology (ESTRO), and the European Society of Pathology (ESP), a fifth group can also be distinguished, which is a combination of markers from the previous groups [5]. This classification has a particularly strong prognostic value in high-risk endometrial cancer where adjuvant therapies are recommended. Furthermore, the introduction of molecular classification may affect the clinical maintenance [6]. If molecular tools are available, the use of this classification in all endometrial cancers is encouraged; however, in low-grade EC with low and intermediate risk, the POLE mutation analysis may be omitted [5].

The Proactive Molecular Risk Classifier for Endometrial Cancer (ProMisE) has been developed as an alternative using immunohistochemical markers instead of sequencing. More research is still needed, as an alternative marker for all TCGA molecular groups has not yet been found [4]. It highlights the heterogeneity of this tumor, which may pose a challenge in endometrial cancer research [7]. Despite the constant discovery of new diagnostic methods and potential therapies, many of the mechanisms involved in cancer initiation and progression remain unclear. Epigenomics, genomics, transcriptomics, proteomics, and metabolomics are the subject of numerous studies as they provide information on gene and protein expression and the mechanisms involved in their regulation. They may allow a better understanding of the phenomena in the course of cancer, the molecular structure of the tumor microenvironment, and the selection of new markers and therapeutic targets [8]. Individual omics have significantly contributed to the identification of epigenetic changes or cancer-specific mutations, but they are somewhat limited as they focus on a single field [9]. Multidimensional analysis can reveal disturbances in several biological systems, giving a broader picture of what happens during tumor initiation and progression [10]. For this reason, multiomics approaches are gaining popularity.

In this paper, we will present the latest findings on the multiomics approaches in endometrial cancer.

## 2. Multiomics Strategies

Studies using multiomics approaches are very valuable, as their results can provide a comprehensive picture of changes taking place in tumors. Multiomics data include genome, transcriptome, proteome, metabolome, and epigenome data. They are generated by methods allowing the determination of gene expression, detection of proteins and their levels [11], studies of DNA and protein interactions [12], assessment of cell proliferation, viability, and migration of even a single cell [13].

The genome is the complete set of DNA of a given organism, knowledge of which can be crucial in disease diagnosis or designing a therapy. The development and application of next-generation sequencing (NGS), including whole genome sequencing (WGS), helps in the comprehensive analysis of genetic material that may reveal important genomic alterations [14]. It is especially important in the case of rare cancers [15], their precise diagnosis and personalized therapy [16], predicting the effectiveness of the therapy [17]. Transcriptome profiling is a widely used approach to analyze the regulation of the expression, function, and structure of genes.

The transcriptome is a set of transcripts present at a specific point in time in a cell, group of cells, or tissue, providing information about both physiological and pathological processes in a specific organism [18]. The study of the transcriptome finds great application in studies aimed at searching for new molecular markers and therapeutic targets. They involve numerous human diseases, including neurological disorders such as chronic pain [19], multiple sclerosis [20], diabetes [21], cardiovascular disease [22], cancers, and their response to treatment [23]. Microarrays and high-throughput RNA sequencing are of great importance, as they rapidly provide a huge amount of data on the processes or organisms under study [24]. Along with the development of new technologies and increasing their availability, the number of studies and new discoveries is growing. Recently, potential biomarkers of colon cancer [25], ovarian cancer [26], hepatocellular carcinoma [27], and endometrial cancer [28] have been selected. Similarly, in the case of the proteome, its analysis reveals detailed information on the activity of individual proteins or protein families, which is valuable in the diagnosis of various diseases, including cancer. The identified proteins can act as diagnostic and prognostic markers [29]. Moreover, the observed changes in protein levels may be the target of therapy [30].

Changes in the degree of DNA methylation, chromatin structure, and post-translational modifications of histones may lead to the development of pathologies, including cancer. Genome-wide epigenomic profiling allows the identification of epigenetic changes in the studied material, which may then become a molecular marker [31]. DNA methylation is one of the most frequently analyzed epigenetic modifications. Changes in its pattern are also important for the development of new treatment strategies, their effectiveness, and the prediction of the end result [32].

Therefore, multiomics approaches generate a great deal of specific data types, such as expression level or nucleotide sequence. To obtain a more complete picture of biological processes and to fully understand observed changes, it is necessary to integrate and analyze these data. For example, deep learning can be used, which will result in improvement in the determination of disease risk, its prevention, or prediction, as well as the development of a personalized treatment strategy [33].

### 2.1. Repositories and Integration Methods

In addition to generating multiomics data, it is also possible to use the available public datasets. Table 1 list the repositories where data from cancer patients, including endometrial cancer, can be found.

The Cancer Genome Atlas is one of the largest genome datasets. It provides information on 33 different types of cancer, including endometrial cancer, for over 20,000 samples. The generated data include high-throughput RNA-Seq, DNA-Seq, miRNA-Seq, DNA methylation, single-nucleotide variant, copy number variation, and reverse phase protein array data. As a result, the database contains information about RNA, DNA, protein profiles, and epigenetic changes along with histopathological and clinical data, which, if properly processed and analyzed, can reveal the mechanisms underlying cancer initiation and progression [34]. Interestingly, the Clinical Proteomic Tumor Analysis Consortium contains datasets generated from the mass spectrometry of cancer samples previously analyzed by TCGA [35].

The Cancer Cell Line Encyclopedia contains datasets generated for nearly 1000 cancer cell lines, including endometrial cancer. Currently, the obtained data concern gene expression, drug sensitivity, whole genome sequencing, RNA-Seq, DNA methylation, reverse phase protein array data, profiling of histones, miRNAs, and metabolites. Therefore, they can be used to identify new molecular markers and understand the response to anti-cancer treatment [36].

In addition to the above-mentioned repositories, there is also the Omics Discovery Index (https://www.omicsdi.org) (accessed on 1 December 2021), which is a collection of datasets from other repositories [37], or TARGET (https://ocg.cancer.gov/programs/target) (accessed on 1 December 2021) for pediatric cancer data [38].

The conducted analyses provide many datasets for individual omics, which indicates the need for their integration in order to obtain a complete picture of the changes occurring in the course of cancer. Interestingly, many studies, although obtaining information on several molecular layers, carry out separate analyses instead of developing a unified model [39]. There are models that treat each layer as a separate entity and include multivariate regression and multi-objective optimization [40]. They are part of multi-layer simultaneous integration, which is also known as meta-dimensional data integration. Carrying out such an analysis requires the prior fusion of data from the examined omics. This method of integration is used in analyses aimed at identifying molecular markers, signaling disruption, or distinguishing cancer subtypes. There is also sequential integration, which is mainly used in the analysis of the influence of one omic on another. As a result, it is possible to better understand the interactions between different levels of gene activity regulation and their importance in oncogenesis [41].

Therefore, the selection of the appropriate data integration and analysis depends on the data type and study objectives. The applied approaches, and thus the methods and algorithms, can also be divided depending on whether they are network or Bayesian. Network approaches are based on known or predicted relationships between variables. They can be determined on the basis of correlation analysis, used, for example, in the Similarity Network Fusion (SNF) method, which allows the identification of the disease subtype and the prediction of its phenotype. Bayesian approaches involve the use of a statistical model in which, using Bayes’ theorem, a posteriori data probability distribution can be computed. An example is the Pathway Recognition Algorithm using Data Integration on Genomic Models (PARADIGM), based on the use of Bayesian factor graphs, which provides information on the degree of signaling pathway activity disruption in patients [40].

### 2.2. Challenges in Multiomics Approaches

As previously mentioned, multiomics approaches generate and analyze large amounts of data. For this reason, there is a risk of making a mistake that may affect the final result. One of the first decisions to be made in multiomics analysis is the selection of the appropriate layers. It is necessary to consider what relationships are important in our study, as well as which omics they relate to, in order to optimally use the obtained data [42]. It is also important for all the analyzed layers to come from a given sample per replicate. Otherwise, it is necessary to use methods that perform calculations based on the aggregation of data [43]. From a technical point of view, the problem may be the preparation and even distribution of material for each omic analysis. In the case of cell culture, an adequate number of cells should be available. On the other hand, if the studied material consists of tissues, attention should be paid to their initial processing, as it will depend on the omic layer [42].

The obtained datasets may also contain measurement errors or noise resulting, for example, from different time scales in the omics layers, as well as the lack of unified data analysis protocols after processing [44]. Moreover, the individual layers contain different types of data and also differ in their number. Such discrepancies can be a problem later in data integration, especially when using machine learning models. It may be necessary to decrease the number of variables to reduce noise and dimensionality, thereby improving performance and computing speed. It is possible by filtering data by applying statistical analysis or removing recursive data. It may also be beneficial to transform the input variables into a new set of variables, which is an accumulation of input features using the Principal Component Analysis (PCA) method. An interesting solution is also to combine all data into one matrix, but it requires earlier reduction of its variables. After such early data integration, deep learning is largely used [45]. A common multiomics approach is also the analysis of single omics, which is followed by data integration. Such a strategy is not difficult and allows the use of easily available tools. The results of each omic analysis can be aggregated into one final result. However, it is worth noting that interactions between omics may be ignored, and in the case of machine learning, individual models do not communicate with each other and thus do not share information [46].

Multiomics approaches also require an infrastructure that will provide adequate computing power as well as the ability to store and share data. Moreover, some omics are much better tested than others, which, apart from technical requirements and costs, is an obstacle to the use of multiomics in routine diagnostics [44]. Large sets of generated data, discrepancies in their number and type, non-paired samples, require taking actions to reduce their complexity. Therefore, it is important to select an appropriate integration method to facilitate data analysis and interpretation.

## 3. Multiomics Approaches in Endometrial Cancer

Multiomics data generated from the same set of samples can provide useful information on the flow of genetic information at multiple levels and thus help unravel the mechanisms underlying the biological state of interest. In the case of endometrial cancer, conducting literature searches on PubMed (https://pubmed.ncbi.nlm.nih.gov) (accessed on 1 December 2021) and Scopus (https://www.scopus.com) (accessed on 3 December 2021) using the following keywords: “endometrial cancer” AND multi-omics, “endometrial cancer” AND multiomics revealed that in the last 5 years, there were several studies on multiomics approaches in this cancer. Original articles in English with human studies and full-text available were taken into account. Studies in which bioinformatics analyses using data from repositories were supplemented with experimental studies were selected for the review. Table 2 summarizes the methods and omics described in the recent endometrial cancer studies. Key findings from these works are also included.

Most of the conducted studies included analysis of the genome, transcriptome, proteome, and also epigenome. An important stage of the analysis was also the use of portals allowing the analysis of multiomics data and their visualization.

### 3.1. Multiomics Data Analysis and Visualization Portals

#### 3.1.1. Oncomine Database

The Oncomine database (http://www.oncomine.org) (accessed on 1 December 2021) is one of the largest microarray datasets, collecting information on over 18,000 microarray experiments. Interestingly, cancer microarray data that are included in the Gene Expression Omnibus (GEO) and Stanford Microarray Database (SMD) are automatically transferred to the Oncomine database, thanks to which the database is constantly expanding. Such data are standardized by researchers involved in this project and can be used to identify genes or signaling pathways that are deregulated in a given cancer or its subtype [55]. Recently, several analyses were carried out using the Oncomine database, which, supplemented with additional studies, allowed the selection of new biomarkers for endometrial cancer.

Gao et al. evaluated the expression of tomoregulin-2 (TMEFF2) to assess its role in endometrial cancer and determine its diagnostic and therapeutic potential. For this purpose, they used TCGA endometrium data, which included 338 blood samples, 354 endometrial cancer tissue samples, and 25 normal endometrial tissues. Their results showed that compared to the control, TMEFF2 DNA copy number was greater in endometrioid EC, serous EC, and mixed EC [47]. The observed overexpression has not been previously described for endometrial cancer but is consistent with the already published results for ovarian cancer, where it is promoted via the mitogen-activated protein kinase (MAPK) and phosphoinositide 3-kinase (PI3K)/protein kinase b (AKT) pathways [56].

Similarly, Geng et al. used the Oncomine database to assess the expression of Zinc finger and BTB domain-containing protein 7A (ZBTB7A). Significant reduction in mRNA expression and DNA copy number variation of ZBTB7A was noted in endometrial cancer compared to the control [49]. This is a valuable observation, as ZBTB7A is considered an oncogene that may constitute a potential therapeutic target. It has already been reported for breast cancer [57], prostate cancer [58], hepatocellular carcinoma [59], and non-small cell lung cancer [60].

In turn, Zou et al. investigated the expression of leucine-rich repeat-containing G-protein coupled receptor 5 (LGR5), somatostatin (SST), prostaglandin D2 synthase (PTGDS), and zinc finger protein 558 (ZNF558). They observed LGR5 overexpression in endometrioid EC and serous EC, as well as SST overexpression in endometrioid EC, serous EC, and mixed EC compared to normal endometrium. However, they did not notice any significant changes in the PTGDS and ZNF558 level [54].

#### 3.1.2. UALCAN Database

The UALCAN database (http://ualcan.path.uab.edu) (accessed on 1 December 2021) uses publicly available TCGA level 3 (processed and ready for high-level analyses) RNA-seq and clinical data from 31 cancer types. It allows the analysis of gene expression in neoplastic and normal tissues as well as in cancer subgroups selected based on the cancer stage, tumor grade, body weight, race, or other features. It is also possible to assess the correlation between the studied expression and patient survival, which may be helpful in identifying new molecular markers [61].

In a recent endometrial cancer study, 581 TCGA tissue samples, including 546 EC samples and 35 normal endometrial samples, were used during analysis. Expression of TMEFF2 [47] and BTG anti-proliferation factor 1 (BTG1) [50] was lower in primary endometrial cancer compared to the healthy endometrium, while significant overexpression was noted for ring finger protein 183 (RNF183) [48]. In turn, the ZBTB7A expression was low in endometrial cancer tissues grouped according to cancer stage, age, and race compared to the control group [49].

Considering the different histological subtypes, the TMEFF2 level was higher in serous EC and mixed EC compared to the control, while the differences observed for endometrioid EC were statistically insignificant [47]. Interestingly, the expression of RNF183 and BTG1 was higher in endometrioid EC compared to the non-endometrioid subtypes. In addition, analyses using the Oncomine database also allowed observing a high expression of RNF183 and BTG1 in TP53-Non-Mutant EC compared to TP53-Mutant EC [48,50].

#### 3.1.3. GEPIA Database

The Gene Expression Profiling Interactive Analysis (GEPIA, http://gepia.cancer-pku.cn) (accessed on 1 December 2021) database is an online tool that uses RNA-Seq data from the TCGA and GTEx projects. Apart from the differential expression analysis of the studied genes, it is also possible to assess the patient’s survival [62].

In the case of RNF183, its high level was noted in endometrial cancer compared to the normal endometrium, which was also confirmed by the analysis using the previously mentioned UALCAN database [48]. This is not the first report regarding endometrial cancer, as Colas et al. have already described the existence of a correlation between RNF183 expression in primary EC and its level in uterine fluid samples [63]. However, this indicates an important role of RNF183 in the course of endometrial cancer and its diagnostic potential, but it would be beneficial to conduct more detailed analyses, similar to studies on colorectal cancer, where it was found that this protein activates the NF-κB–IL-8 axis, which leads to increased proliferation and metastasis [64].

The reduced ZBTB7A level in endometrial cancer was observed in analyses using the UALCAN and Oncomine databases and was validated with the GEPIA database [49]. In addition, the overexpression of LGR5 and SST in endometrial cancer reported with the Oncomine database was also confirmed with GEPIA. Interestingly, the expression of ZNF558 and PTGDS was significantly decreased in endometrial cancer in this analysis [54]. An analysis was also performed for homeobox B9 (HOXB9), and an increase in its expression in endometrial cancer samples compared to normal endometrium was observed, suggesting that HOXB9 may be a prognostic marker for EC [51]. Similar conclusions were drawn in non-small cell lung cancer [65], breast cancer [66], gastric cancer [67], and laryngeal squamous cell carcinoma [68].

#### 3.1.4. Kaplan–Meier Plotter Database

The Kaplan–Meier (KM) Plotter (http://kmplot.com/analysis) (accessed on 1 December 2021) is a tool that allows researchers to perform a survival analysis and determine the prognostic value based on the expression of the studied gene [69].

Endometrial cancer was divided into groups with low and high gene expression. One analysis showed that a high positive expression of TMEFF2 was associated with a shortened overall survival (OS). It should be noted that the study included 75 endometrial cancer patients, 20 of whom died and four were lost to follow-up. In addition, age, FIGO stage, depth of myometrial invasion, lymph node metastasis, ER status, and PR status were all correlated with poor prognosis [47].

On the other hand, Zou et al. revealed that the mean OS was significantly lower in the group with low PTGDS expression. In total, 36 patients died and 13 were lost to follow-up of 87 patients with endometrial cancer. A multivariate Cox analysis was performed, which indicated the following independent risk factors affecting the prognosis: low PTGDS expression, advanced cancer stage, and deeper myometrial invasion [54].

Another study based on RNA-seq data reported that 542 patients had high RNF183 expression, which was associated with favorable overall survival and progression-free survival [48]. It was similar in the study by Li et al., where higher OS was noted in patients with high BTG1 expression [50]. In turn, high expression of HOXB9 and E2F3 correlated with a shorter survival time [51].

In the case of the ADP-ribosylation factor (ARF)/ARF-like protein (ARL) family, it was observed that a high expression of ARL4D, ARL1, ARF1, and SAR1B in endometrial cancer is associated with better prognosis, while a high expression of ARL4C, ARL2, ARL10, ARL16, and ARL14 promotes worse survival. Interestingly, the combination of ARL4C with CDK6 or MYC allowed noticing that high expression of the studied genes is associated with shorter survival times. This indicates that the use of a combination of two genes, in this case ARL4C and CDK6 or MYC, enhances survival predictions [55].

#### 3.1.5. TIMER Database

Tumor Immune Estimation Resource (TIMER, http://cistrome.shinyapps.io/timer) (accessed on 1 December 2021) is a tool that allows the analysis of the tumor-infiltrating immune cells (TIIC), including B cells, CD4 T cells, CD8 T cells, macrophages, neutrophils, and dendritic cells, in studied cancers. It is based on data from over 10,000 samples for 32 types of TCGA cancers [70].

In the case of RNF183, the analysis showed its high level in endometrial cancer compared to normal endometrium, which is consistent with the previously used tools. Further analysis showed that there is a negative correlation between RNF183 expression and tumor purity, infiltrating levels of CD4+ T cells, neutrophils, and dendritic cells. In endometrial cancer, infiltrating levels of CD8+ T cells, macrophages, and dendritic cells have also been reported to be affected by the RNF183 copy number variation [48].

Similarly, analysis of the ZBTB7A gene showed its reduced expression, which was previously determined with other databases. The observed low ZBTB7A expression resulted in unfavorable overall survival and disease-free survival (DFS) in patients with endometrial cancer. Moreover, a correlation was also noted between ZBTB7A expression and infiltrating levels of CD8+ T cells, neutrophils, and dendritic cells [51].

TIMER database analysis also showed a positive correlation between PTGDS expression and infiltration levels of B cells, CD4+ T cells, and macrophages. In contrast, the PTGDS copy number variation correlated with infiltration levels of B cells, CD8+ T cells, macrophages, and dendritic cells. Further analysis revealed that poor prognosis favors low B cell and CD8+ T cell infiltration [54].

TIMER database can also be used to evaluate the differential expression between endometrial cancer and normal endometrium. It was observed that ARFRP1, ARL1, ARL10, ARL13B, ARL15, ARL2, ARL3, ARL4A, ARL4D, ARL5A, ARL5C, ARL8B, and TRIM23 showed low expression in endometrial cancer compared to the control, while ARF1, ARF3, ARF4, ARF5, ARF6, ARL11, ARL14, ARL16, ARL4C, ARL5B, ARL8A, ARL9, and SAR1B were overexpressed [53].

#### 3.1.6. LinkedOmics Database

The LinkedOmics database (http://www.linkedomics.org) (accessed on 1 December 2021) uses datasets of over 11,000 patients and 32 cancer types from the TCGA project. For breast cancer, colorectal cancer, and ovarian cancer, the database also includes mass spectrometry-based proteomics data generated by the Clinical Proteomic Tumor Analysis Consortium. The database module allows the identification of genes related to the studied gene based on Pearson correlation coefficient analysis, visualization of results as heat map, volcano plot, or scatter plot. Moreover, through the gene set enrichment analysis (GSEA), it is possible to analyze gene ontology (GO) categories and Kyoto Encyclopedia of Genes and Genomes (KEGG) pathways [71].

The analysis using the LinkedOmics database was carried out for 176 patients with endometrial cancer. Based on the volcano plot, it has been observed that 2307 genes are correlated with TMEFF2, most of which are positively correlated. The statistical scatter plots revealed positive correlations between the expression of TMEFF2 and LIM homeobox 8 (LHX8), paired box gene 3 (PAX3), ASXL transcriptional regulator 3 (ASXL3). Then, in order to identify the targets of TMEFF2 in endometrial cancer, GSEA was performed. This analysis revealed kinase, miRNA, and transcription factor target networks that are positively correlated with TMEFF2. Targets included polo-like kinase 2 (PLK2), protein kinase X-linked (PRKX), protein kinase cAMP-activated catalytic subunits gamma (PRKACG) and beta (PRKACB), cyclin-dependent kinase 5 (CDK5), miR-200b, miR-200c, miR-429, miR-190, miR-448, miR-141, miR-200a, and miR-9 [47].

The analysis performed for the BTG1 gene revealed strong correlations between the expression of BTG1 and SH3 domain-binding glutamic acid-rich-like protein 3 (SH3BGRL), protein-L-isoaspartate O-methyltransferase domain-containing protein 1 (PCMTD1), and tetmethylcytosine dioxygenase 2 (TET2). Moreover, taking into account the top 50 gene sets related to BTG1, their functions include the regulation of cell cycle, apoptosis, and carcinogenesis [50].

In the case of RNF183, the number of genes correlated with its expression was much greater and amounted to almost 20,000. GSEA and GO term analysis showed that differentially expressed genes correlated with RNF183 are associated with stimulus response as well as metabolic and biological regulation. In addition, the KEGG pathway analysis revealed enrichment in, among others, IL-17 and PPAR signaling pathways, fatty acid degradation, or drug metabolism [48].

Similarly for ZBTB7A, approximately 20,000 genes correlated with its expression. The implementation of GSEA allowed selecting the following targets of ZBTB7A: polo-like kinase 1 (PLK1), cyclin-dependent kinase 1 (CDK1) and 2 (CDK2), Aurora kinase B (AURKB), tumor-associated macrophages (ATM), miR-212, miR-132, miR-499, miR-202, miR-485-3P, miR-522, and E2F family [49].

Wan et al. performed GO and KEGG enrichment analysis that showed a wide range of processes and signaling pathways associated with HOXB9, which included regulation of cell cycle, metabolism, or P53 and p38 MAPK pathways [51]. In a study of ARF/ARL family members, KEGG enrichment analysis showed that ARL4C overexpression may be associated with cell adhesion and cell cycle signaling pathways. Interestingly, the regulation of cell adhesion was positively correlated with high ARL4C expression. Similarly, cell cycle regulation was enriched in the ARL4C highly expressing group [53].

Almost 20,000 genes were correlated with PTGDS expression in endometrial cancer, and according to GSEA and GO term analysis, they were mainly associated with immunological processes, including regulation of the inflammatory response, leukocyte activation, or adaptive immune response. On the other hand, processes related to the activity of the cell cycle, e.g., mRNA processing and phase transition, were inhibited. KEGG pathway analysis showed that genes positively correlated with PTGDS expression are associated with chemokine signaling pathways, adhesion molecules, as well as cytokine-cytokine receptor interactions [54].

#### 3.1.7. miRNA Analysis

Given the significant role of miRNAs in the regulation of gene expression, the analysis of miRNAs can provide a lot of valuable information in the search for new molecular markers or therapeutic targets [72].

In recent endometrial cancer research, the LinkedOmics database has been used to find networks of miRNA targets, as was already described in detail [47,48]. In addition, Li et al. used the miRDB database (http://mirdb.org) (accessed on 1 December 2021) in their study, as it allows the prediction of miRNA targets and functional annotation [50,73]. The aim of the study was to select miRNAs that can bind to BTG1. Analysis in miRDB showed that there are 58 such miRNAs. Among them, the highest target scores were obtained by miR-513a-5p, miR-548t-5p, miR-302a-3p, miR-302c-3p, miR-580-3p, miR-302d-3p, and miR-302b-3p.

### 3.2. Validation of Bioinformatics Analysis

Bioinformatics analysis provides a lot of information on the expression of the studied genes and potential interactions between genes and proteins, and it helps identify signaling pathways associated with particular genes, as well as target miRNAs. However, these results refer to existing datasets, mainly of TCGA cancers, that are used by the databases; therefore, many researchers validate them by performing experiments using tissue samples and/or cell cultures.

#### 3.2.1. Immunohistochemistry

The assessment of TMEFF2 protein level was performed with the immunohistochemical streptavidin-peroxidase method. The analysis was conducted on 135 endometrial samples, including 36 cases of normal endometrium, 24 cases of atypical endometrial hyperplasia, and 75 cases of endometrial cancer. The staining showed that TMEFF2-positive expression rates were higher in EC and atypical endometrial hyperplasia compared to normal endometrium. In addition, the positive expression rates of TMEFF2 were significantly higher in EC compared to the atypical hyperplasia group. In further analysis, it was observed that as the degree of cancer differentiation decreased, the high expression rate of TMEFF2 gradually increased [47].

Expression at the protein level was also determined for HOXB9 in 15 normal endometrial samples, 21 atypical endometrial hyperplasia, and 88 EC samples. The HOXB9 protein level correlated with histological grade as well as lymph node metastasis [51]. In the case of the TTK protein, its expression was determined in 33 samples of normal endometrium, 16 endometrial hyperplasia without atypia, 21 endometrial atypical hyperplasia, and 63 EC. Staining showed that TTK is overexpressed in endometrial cancer. There was no correlation between the TTK level and the histological grade, myometrium, and lymph node invasion. Positive correlation occurred between TTK expression and the ascending FIGO stage [62]. PTGDS protein concentration was assessed in 116 samples, including 12 normal endometrial tissue samples, 17 atypical endometrial hyperplasia, and 87 EC samples. The positive and highly positive expression rates of PTGDS were lower compared to the atypical hyperplasia group and normal endometrium [54].

Human tissue microarrays were used to assess ARL4C protein levels in endometrial cancer. The analysis was performed for 34 EC samples and 26 samples of normal endometrium. Staining showed that ARL4C expression is higher in EC compared to normal endometrium. Interestingly, the ARL4C level did not correlate with the histological grade and FIGO stage [53].

#### 3.2.2. Cell Cultures

Cell-based assays were conducted in seven out of the eight studies included in this review. The following cell lines were used: Ishikawa, HEC-1A, HEC-1B, RL95-2, AN3CA, and KLE. Depending on the purpose of the study, cells were transfected with siRNA, as well as cell proliferation, wound healing, and Transwell assays were performed. Detection of the expression of individual genes was performed by Western blot, qRT-PCR, or qPCR.

In the case of TMEFF2, Western blot revealed that the level of this protein was higher in the Ishikawa cell line compared to HEC-1A and HEC-1B. It was also observed that the expression of this protein in Ishikawa cells was inhibited by RNA interference. Further analyses showed that the mentioned inhibition of TMEFF2 expression resulted in a decrease in proliferation capacity, migration, and invasion by Ishikawa cells. The levels of EMT-related proteins, including E-cadherin, vimentin, matrix metalloproteinase-2 (MMP2), and matrix metalloproteinase-9 (MMP9) were also assessed by Western blot and immunohistochemistry in this study. It was found that the inhibition of TMEFF2 expression led to an increase in E-cadherin and a decrease in vimentin, MMP2, and MMP9 [47].

In the study of RNF183, it was revealed that it positively correlates with ESR1 expression. Silencing of RNF183 with siRNA in an ERα-positive Ishikawa cell line allowed observing a decreased level of ESR1 with qRT-PCR. Interestingly, ERα knockdown had little effect on the level of RNF183 [48]. In turn, high levels of ZBTB7A in Ishikawa cells inhibit their proliferation and migration as well as repress E2F4 level [49].

Studies have also shown that BTG1 overexpression can inhibit the proliferation and invasion of Ishikawa and HEC-1A cells as well as promote apoptosis. On the other hand, ARL4C knockdown in Ishikawa and HEC-1A cells resulted in a decreased ability to migrate and invade. The effect of BTG1 expression on EMT was assessed by determining the expression of E-cadherin, vimentin, and N-cadherin with Western blot. A high level of E-cadherin and decreased N-cadherin and vimentin levels were associated with BTG1 overexpression [50].

In the case of HOXB9, its silencing in the Ishikawa cell line had no effect on cell proliferation and colony number decrease. However, HOXB9 knockdown affected the migration ability of Ishikawa and RL95-2 cells. Interestingly, a wound-healing assay showed that HOXB9 overexpression accelerated Ishikawa cell migration. The study also revealed that HOXB9 knockdown resulted in a reduction of E2F3 protein levels. Additionally, the silencing of E2F3 reduced the migration of Ishikawa cells [51].

The expression of TTK was determined by qRT-PCR in the AN3CA, HEC-1-A, HEC-1-B, RL95-2, and KLE cell lines. TKK was overexpressed in all five endometrial cancer cell lines, which was fully confirmed by Western blot. Further analyses showed that the silencing of TTK in AN3CA and HEC-1-B cells by siRNA decreased cell proliferation and induced apoptosis [52].

## 4. Concluding Remarks

Multiomics studies provide extremely important and detailed information on topics related to the molecular basis of cancer, potential molecular markers, and therapeutic targets. The volume of available data may seem overwhelming, but there are many tools to handle it in an accessible way. Bioinformatics databases are constantly updated, which expands our research capabilities. Easy access to databases and the ability to validate the results with the use of independent tools encourage more advanced analyses. A single omic strategy provides useful knowledge, but techniques that allow the simultaneous analysis of multiple omics seem to be promising. The observation that the expression of a given gene changes seems to be insufficient when we take into account the complexity of the mechanisms accompanying cancer development. The use of bioinformatics databases can allow understanding the correlation and interactions between genes and/or proteins, identify signaling pathways in which they participate, or select targets for miRNAs.

The large number of diagnosed endometrial cancers emphasizes the need for further investigation of the mechanisms involved in the initiation and progression of this cancer. Analyses based on the multiomics strategy can help identify markers for the early detection of endometrial cancer or potential therapeutic targets. The works analyzed in this review provided some important findings.

It was observed that TMEFF2 expression was increased in endometrial cancer, and its silencing inhibited EMT and the activation of MAPK and PI3K pathways, which suggest that TMEFF2 can help diagnose and treat endometrial cancer [47]. Similarly for RNF183, its association with ERα may be a marker in ERα-positive endometrial cancer [48]. In the case of ZBTB7A, its reduced expression in endometrial cancer is associated with unfavorable overall survival and disease-free survival. In addition, ZBTB7A participates in the regulation of tumor immunity, making it a promising EC biomarker [49].

BTG1 could be another potential endometrial cancer biomarker. It is considered a suppressor gene and inhibits proliferation, migration, as well as the EMT process in the EC [50]. Recent studies have also shown the overexpression of HOXB9 in endometrial cancer, which could potentially be a prognostic marker and a therapeutic target [51]. TTK, acting as an oncogene, can promote cell proliferation and therefore can be used as a therapeutic target for EC [52].

It has also been demonstrated that ARL4C belonging to the ARF/ARL family can induce the proliferation, migration, and invasion of endometrial cancer. This is possible by regulating cell adhesion and the cell cycle; therefore, ARL4C is considered to be a promising target in the treatment of endometrial cancer [53]. Moreover, it has also been reported that LGR5, SST, ZNF558, and PTGDS may be involved in the development and progression of endometrial cancer. Interestingly, it is suggested that PTGDS can act as a suppressor gene. It is associated with immunological processes and thus can be a therapeutic target for EC or its biomarker [54].

Endometrial cancer studies carried out in recent years with the use of multiomics strategies allowed for the selection of a number of molecular markers and therapeutic targets, thus enriching the current knowledge about endometrial cancer and the mechanisms related to its development.

## Figures and Tables

**Table 1:** Publicly available repositories of multiomics cancer data, including endometrial cancer data.

Repository Name	Web Link	Data Available
The Cancer Genome Atlas (TCGA) [34]	https://cancergenome.nih.gov (accessed on 1 December 2021)	RNA-Seq, DNA-Seq, miRNA-Seq, DNA methylation, SNV, CNV, RPPA
Clinical Proteomic Tumor Analysis Consortium (CPTAC) [35]	https://cptac-data-portal.georgetown.edu (accessed on 1 December 2021)	proteomics data corresponding to the TCGA samples
Cancer Cell Line Encyclopedia (CCLE) [36]	https://portals.broadinstitute.org/ccle (accessed on 1 December 2021)	gene expression, drug sensitivity data, WGS, histone profiling, RNA-Seq, DNA methylation, miRNA profiling, metabolite profiling, RPPA

SNV, single-nucleotide variant; CNV, copy number variation; RPPA, reverse phase protein array.

**Table 2:** Analysis methods and omics in endometrial cancer studies, including key findings.

Reference	Methods	Omics	Key Findings
Gao et al. [47]	bioinformatics, immunohistochemistry, cell-based assay, Western blot	genomics, transcriptomics, epigenomics, proteomics	TMEFF2 as target for early diagnosis and EC treatment
Geng et al. [48]	bioinformatics, cell-based assay, qPCR, Western blot	genomics, transcriptomics, proteomics	RNF183 as prognostic and early diagnostic indicator for EC
Geng et al. [49]	bioinformatics, cell-based assay, qPCR	genomics, transcriptomics, epigenomics proteomics	ZBTB7A as prognostic biomarker for EC
Li et al. [50]	bioinformatics, cell-based assay, qRT-PCR, Western blot	genomics, transcriptomics, epigenomics proteomics	BTG1 as prognostic biomarker for EC
Wan et al. [51]	bioinformatics, immunohistochemistry, cell-based assay, qPCR	genomics, transcriptomics, proteomics	HOXB9 is correlated with EC cell migration and promotes its progression
Zhang et al. [52]	immunohistochemistry, cell-based assay, qRT-PCR, Western blot	genomics, transcriptomics, proteomics	TTK as therapeutic target for EC
Zhang et al. [53]	bioinformatics, immunohistochemistry, cell-based assay, qPCR	genomics, transcriptomics, proteomics	ARF/ARL family genes as prognostic biomarkers for EC
Zou et al. [54]	bioinformatics, immunohistochemistry	genomics, transcriptomics, proteomics	LGR5, SST, ZNF558, and PTGDS participate in the development of EC; PTGDS as biomarker and therapeutic target for EC

TMEFF2, tomoregulin-2; RNF183, ring finger protein 183; ZBTB7A, zinc finger and BTB domain-containing protein 7A; BTG1, BTG anti-proliferation factor 1; HOXB9, homeobox B9; TTK, TTK protein kinase; ARF/ARL, ADP-ribosylation factor/ARF-like protein; LGR5, leucine-rich repeat-containing G-protein coupled receptor 5; SST, somatostatin; ZNF558, zinc finger protein 558; PTGDS, prostaglandin D2 synthase.

## Data Availability

The data used to support the findings of this study are included in the article.
